# The structure of an authentic spore photoproduct lesion in DNA suggests a basis for recognition

**DOI:** 10.1107/S1399004713032987

**Published:** 2014-02-15

**Authors:** Isha Singh, Yajun Lian, Lei Li, Millie M. Georgiadis

**Affiliations:** a Department of Biochemistry and Molecular Biology, Indiana University School of Medicine, Indianapolis, IN 46202, USA; b Department of Chemistry and Chemical Biology, Indiana University–Purdue University at Indianapolis, Indianapolis, IN 46202, USA

**Keywords:** spore photoproduct, DNA, host–guest approach

## Abstract

The spore photoproduct lesion (SP; 5-thymine-5,6-dihydro­thymine) is the dominant photoproduct found in UV-irradiated spores of some bacteria such as *Bacillus subtilis*. Upon spore germination, this lesion is repaired in a light-independent manner by a specific repair enzyme: the spore photoproduct lyase (SP lyase). In this work, a host–guest approach in which the N-terminal fragment of Moloney murine leukemia virus reverse transcriptase (MMLV RT) serves as the host and DNA as the guest was used to determine the crystal structures of complexes including 16 bp oligo­nucleotides with and without the SP lesion at 2.14 and 1.72 Å resolution, respectively. In contrast to other types of thymine–thymine lesions, the SP lesion retains normal Watson–Crick hydrogen bonding to the adenine bases of the complementary strand, with shorter hydrogen bonds than found in the structure of the undamaged DNA. However, the lesion induces structural changes in the local conformation of what is otherwise B-form DNA. The region surrounding the lesion differs significantly in helical form from B-DNA, and the minor groove is widened by almost 3 Å compared with that of the undamaged DNA. Thus, these unusual structural features associated with SP lesions may provide a basis for recognition by the SP lyase.

## Abstract 2

The structure of a spore photoproduct lesion in duplex DNA is described.

## 1. Introduction   

Exposure to UV light can lead to a number of modifications in an organism’s DNA. Some of these modifications involve the formation of pyrimidine dimers such as cyclobutane pyrimidine dimers (CPDs), pyrimidine (6–4) pyrimidone photoproduct [(6–4) PD] and Dewar isomer lesions, which arise from a UV-A/B-induced 4π sigmatropic rearrangement of (6–­4) PD lesions (Yang, 2011 ▶; Douki & Cadet, 2003 ▶; Glas *et al.*, 2010 ▶). A unique thymine dimer, 5-thyminyl-5,6-dihydro­thymine or spore photoproduct (SP) lesion (Donnellan & Setlow, 1965 ▶; Douki & Cadet, 2003 ▶; Moeller *et al.*, 2007 ▶), is formed in spores of bacteria such as *Bacillus subtilis* in response to UV irradiation. The spore DNA is protected by the low cell-hydration level as well as the presence of high levels of Ca^2+^ dipicolinic acid and small acid-soluble spore proteins (Setlow, 1992 ▶, 2006 ▶). This unique photoproduct is formed preferentially in the 5*R*-SP configuration in dehydrated DNA (A-form) found in spores (Douki *et al.*, 2003 ▶; Chandra *et al.*, 2009 ▶; Mantel *et al.*, 2008 ▶; Lin *et al.*, 2011 ▶). Moreover, spores contain a unique repair enzyme called spore photoproduct lyase that repairs these spore photoproduct lesions on germination following hydration of the spore. This unique spore photoproduct lesion along with the direct repair enzyme spore photoproduct lyase offers an evolutionary advantage to spore resistance and survival (Buis *et al.*, 2006 ▶; Chandor *et al.*, 2006 ▶; Slieman *et al.*, 2000 ▶). In general, lesions caused by exposure to UV light hinder the ability of DNA to replicate and, if unrepaired, hold mutagenic potential (Gentil *et al.*, 1996 ▶).

Many organisms have evolved the ability to repair these lesions. Light-dependent enzymes called CPD photolyase and (6–4) photolyase repair CPDs and (6–4) PDs, respectively (Todo, 1999 ▶). CPD photolyases are found in organisms ranging from bacteria, fungi, plants and invertebrates to many vertebrates, while (6–4) photolyases occur in *Drosophila*, silkworms, frogs and rattlesnakes (Sancar, 1996 ▶; Yasui *et al.*, 1994 ▶). No photolyases are present in humans; however, mismatch DNA-repair and nucleotide-excision repair pathways are present in humans to repair pyrimidine dimers (Thoma, 1999 ▶). Both (6–4) photolyases and CPD photolyases have the same overall fold, with an α/β domain and FAD-binding helical domain (Maul *et al.*, 2008 ▶; Park *et al.*, 2002 ▶), and use the photon energy from near-UV or blue light to catalyze the repair of their respective photoproducts. The Dewar isomer lesions are repaired by (6–4) photolyases through a photoinduced electron-transfer mechanism (Mees *et al.*, 2004 ▶; Park *et al.*, 2002 ▶; Benjdia, 2012 ▶; Glas *et al.*, 2009 ▶). SP lyase, on the other hand, repairs the spore photoproduct through a radical-based mechanism in a light-independent manner involving its [4Fe–4S] cluster and an *S*-adenosyl-l-­methionine (SAM) cofactor (Heil *et al.*, 2011 ▶). The crystal structures of photolyases and the SP lyase suggest a common mechanism for the repair of these photoproducts. Based on the crystal structure of the SP lyase with a dinucleoside SP lesion mimic and SAM bound to the active site, it has been proposed that, like photolyases, the SP lyase fully opens the DNA at the site of the lesion followed by flipping out of the dimer by almost 180° into the active site of the enzyme (Benjdia, 2012 ▶).

Structural changes resulting from the formation of these lesions in duplex DNA lead to their recognition and repair by their respective lyases (Maul *et al.*, 2008 ▶; Benjdia *et al.*, 2012 ▶). The crystal structure of duplex DNA containing the CPD lesion suggests that the dimer bends the DNA by 30° and also widens the minor groove (Park *et al.*, 2002 ▶). The (6–4) PD, on the other hand, induces the loss of base-stacking interactions and distorts the double helix significantly more than the CPD lesion (Glas *et al.*, 2009 ▶). However, the structural changes in double-stranded DNA induced by the intrastrand spore photoproduct lesion are not well understood. In the structure of DNA containing an SP mimic in complex with *B. stearo­thermophilus* DNA polymerase I, the 5*R*-SP lesion was found to form normal Watson–Crick hydrogen bonds (Heil *et al.*, 2011 ▶). However, the extensive contacts between the DNA and polymerase in this structure preclude analysis of the structural changes induced by the lesion within duplex DNA. Thus, prior to this work there was no basis for understanding the structural features of duplex DNA containing an authentic SP that might be recognized by SP lyase.

Here, we present crystal structures of the spore photoproduct within the context of a normal phosphodiester backbone and of the same DNA sequence lacking the SP lesion determined at 2.14 and 1.72 Å, respectively, by using the host–guest approach developed in the Georgiadis laboratory (Coté *et al.*, 2000 ▶). Comparative analysis of the two structures provides insights into the structural features associated with the spore photoproduct within B-form DNA and suggests a basis for understanding how the spore photoproduct lyase recognizes the lesion.

## 2. Materials and methods   

### 2.1. Preparation of an SP-containing oligonucleotide   

The 16-mer deoxyribooligonucleotide (5′-ATCCGttAT­AACGGAT-3′) containing the spore photoproduct thymine dimers, represented by ‘tt’ in the sequence (SP DNA), was synthesized using the SP phosphoramidite developed by the Li laboratory and solid-phase DNA synthesis using an ABI394 DNA/RNA synthesizer as described previously (Jian & Li, 2013 ▶). The resulting 16-mer oligonucleotide was purified by HPLC using a Waters (Milford, Massachusetts, USA) Breeze HPLC system with a 2489 UV–visible detector at 260 nm. An XBridge OST C18 column (2.5 µm, 4.6 × 50 mm) was equilibrated with 5% acetonitrile in 0.1 *M* TEAA buffer at pH 7.0 (buffer *A*), and compounds were eluted with an ascending gradient (0–35%) of buffer *B* composed of 70% buffer *A* and 30% acetonitrile at a flow rate of 1 ml min^−1^ in 15 min. The purified oligonucleotide was further desalted by HPLC using H_2_O (buffer *A*) and acetonitrile (buffer *B*) as the elution solvents. The product was then dried by lyophilization and saved for X-ray crystallographic studies.

### 2.2. Crystallization and data collection   

The N-terminal fragment (amino-acid residues 24–278) of Moloney murine leukemia virus reverse transcriptase (MMLV RT) was purified as described previously (Sun *et al.*, 1998 ▶). In brief, the protein eluted from an Ni–NTA affinity column was loaded onto an S Sepharose column. This was followed by cleavage of the 6×histidine tag with thrombin and again subjecting the protein to S Sepharose ion-exchange chromatography to remove the tag. Finally, the protein was concentrated to 2 m*M* in 50 m*M* MES pH 6.0, 0.3 *M* NaCl, 1 m*M* DTT for further experiments. The 16-mer DNA oligonucleotide with the same sequence but without the thymine dimer (5′-ATCCGTTATAACGGAT-3′) was synthesized and desalted using gel-filtration chromatography by Midland Certified Reagent Company (Midland, Texas, USA). The oligonucleotides were resuspended in buffer consisting of 10 m*M* HEPES pH 7.0, 10 m*M* MgCl_2_ to give a final concentration of 2.5 m*M* duplex. Prior to crystallization studies, the DNA oligonucleotides at 5 m*M* were incubated at 70°C for 10 min and allowed to cool gradually at room temperature to allow the annealing of complementary strands to form the 16-mer duplex.

Protein–DNA crystals were obtained using 1 µl each of microseeds and a 1:1.5 ratio of protein (0.46 m*M*):DNA (0.71 m*M*) solution in vapor-diffusion hanging drops with a reservoir solution consisting of 7% PEG 4000, 5 m*M* mag­nesium acetate, 50 m*M* *N*-(2-acetamido)iminodiacetic acid (ADA) pH 6.5 in the case of SP-DNA, while 9% PEG 4000, 5 m*M* magnesium acetate, 50 m*M* ADA pH 6.5 was used for the non-SP-containing DNA sequence. The protein–SP DNA crystals were stabilized in 9% PEG 4000, 5 m*M* magnesium acetate, 100 m*M* HEPES pH 7.5, 20% ethylene glycol. The cryosoaking solution for the non-SP DNA crystals consisted of 11% PEG 4000, 5 m*M* magnesium acetate, 100 m*M* HEPES pH 7.5, 20% ethylene glycol. Data for the protein–SP DNA crystal were collected to Bragg spacings of 2.14 Å on a Bruker X8 Prospector (Bruker Corporation, Billerica, Massachusetts) with Cu *K*α radiation at 100 K using an Oxford Cryosystem. Data were integrated using *SAINT* and scaled with *SADABS*. The space-group determination and data statistics were calculated using the *XPREP* package. For the protein–DNA (non-SP) complex, data were collected to 1.72 Å resolution on SBC beamline 19-ID of the Advanced Photon Source (APS), Argonne, Illinois, USA (λ = 0.97903 Å) and were processed using the *HKL*-2000 package. Both crystals are orthorhombic, belonging to space group *P*2_1_2_1_2 (see Table 1 ▶ for data-processing statistics and unit-cell parameters).

### 2.3. Structure determination and refinement   

Initial molecular-replacement phases were obtained for the protein–SP DNA structure using the N-terminal fragment of MMLV RT (PDB entry 1ztw; Goodwin *et al.*, 2005 ▶) as the search model in *Phaser* (McCoy *et al.*, 2007 ▶). The model was subjected to rigid-body and then restrained least-squares refinement in *REFMAC* (Murshudov *et al.*, 2011 ▶), and was subsequently refined in *PHENIX* (Adams *et al.*, 2010 ▶) using restrained least-squares refinement to obtain unbiased electron density for the DNA. Following placement and refinement of the first shell of waters, the first three base pairs of the DNA were modelled using *Coot* (Emsley *et al.*, 2010 ▶) and then refined with *PHENIX* (Adams *et al.*, 2010 ▶). The remainder of the DNA model was completed in iterative cycles of model adjustment in *Coot* and crystallographic refinement in *PHENIX*. The final *R*_work_ and *R*_free_ for the refined protein–SP DNA model were 20.1 and 23.9%, respectively (Table 1 ▶).

For data collected from protein–DNA crystals lacking the TT lesion, initial phases were obtained by molecular replace­ment with *MOLREP* (Vagin & Teplyakov, 2010 ▶), with the refined protein model from the protein–SP DNA complex serving as the search model. The DNA model was built using *Coot* (Emsley *et al.*, 2010 ▶), and crystallographic refinement was performed using restrained least-squares refinement in *REFMAC* (Murshudov *et al.*, 2011 ▶) and *PHENIX* (Adams *et al.*, 2010 ▶) as described above for the protein–SP DNA complex. Subsequent refinement of the protein–non SP DNA model yielded *R*_work_ and *R*_free_ values of 21.12 and 23.40%, respectively (Table 1 ▶). As one asymmetric unit of the crystals contains only eight base pairs of duplex DNA, the intact 16-­mer duplexes were generated by symmetry in *Coot* (Emsley *et al.*, 2010 ▶). Coordinates have been deposited with the PDB as entries 4m94 and 4m95 for the SP and non-SP structures, respectively.

### 2.4. Structural analysis   

The DNA structures of the 16-mer duplexes were analyzed using 3*DNA* (Colasanti *et al.*, 2013 ▶; Lu & Olson, 2003 ▶, 2008 ▶). 3*DNA* uses El Hassan and Calladine’s algorithm to calculate the major-groove and minor-groove widths, where each di­nucleotide step is assigned a groove width based on simple cross-strand P–P distances (El Hassan & Calladine, 1998 ▶). The refined groove-width values, which also take into account the directions of the sugar phosphate backbone, were used to plot graphs of the differences in major-groove and minor-groove widths. Contact areas between bases within the SP lesion and the corresponding thymine bases in the non-SP structure were calculated using *NACCESS* (Hubbard & Thornton, 1993 ▶).

## 3. Results   

### 3.1. Overall structure   

The host–guest system used in this study employs the co-crystallization of a 16-base-pair DNA oligonucleotide duplex (guest) with the protein (host), the N-terminal fragment of the Moloney murine leukemia virus reverse transcriptase (MMLV RT), which includes the fingers and palm domains (Coté *et al.*, 2000 ▶). The fingers domain of MMLV RT interacts with the 3′-­OH end of one strand as well as the minor-groove base and sugar atoms of the terminal three base pairs, resulting in several hydrogen bonds and van der Waals contacts (Coté & Georgiadis, 2001 ▶; Coté *et al.*, 2000 ▶, 2003 ▶; Najmudin *et al.*, 2000 ▶). The host–guest system has several important features, two of which are relevant to this work. Firstly, different DNA oligonucleotides are all analyzed within the same crystal lattice and are therefore subject to the same crystalline environment (see Table 1 ▶ for unit-cell parameters). Secondly, the central 10 bp of the 16 bp duplex DNA are free of interactions with the protein or other DNA molecules, allowing the nucleic acid to adopt a structure dictated by its sequence. In previous work, we used this host–guest system to analyze the properties of the 5′-CA dinucleotide step that is recognized and processed by integrase (PDB entries 2fvp, 2fvq, 2fvr and 2fvs) and found that independent of its position within the central 10 bp, the CA dinucleotide step in these structures had similar structural properties, including a positive roll angle and a negative slide value (Montaño *et al.*, 2006 ▶).

**Figure 1:**
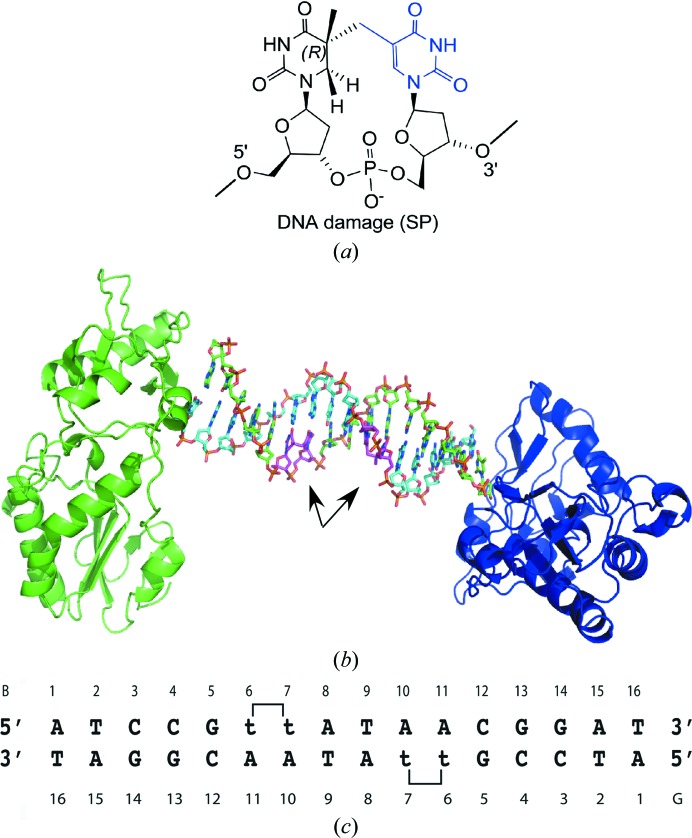
Crystal structure of the SP-containing duplex 16-mer oligonucleotide in a host–guest complex. (*a*) The chemical structure of the spore photoproduct is shown. (*b*) The host–guest complex includes two protein molecules, shown in a cartoon rendering with one molecule in green and the other in blue, and one 16-base-pair duplex, shown in a stick rendering with one strand coloured with C atoms in green, N atoms in blue, O atoms in red and P atoms in orange and the other with C atoms in cyan, N atoms in blue, O atoms in red and P atoms in orange. The asymmetric unit of the crystal includes one protein molecule and eight base pairs of duplex DNA. The SP lesions are shown in magenta and are indicated by arrows. (*c*) The sequence of the SP-containing DNA used for this study is shown with its numbering scheme. The thymines involved in the SP lesion are indicated as ‘t’. The same sequence lacking the SP lesion was also crystallized and analyzed.

**Figure 2:**
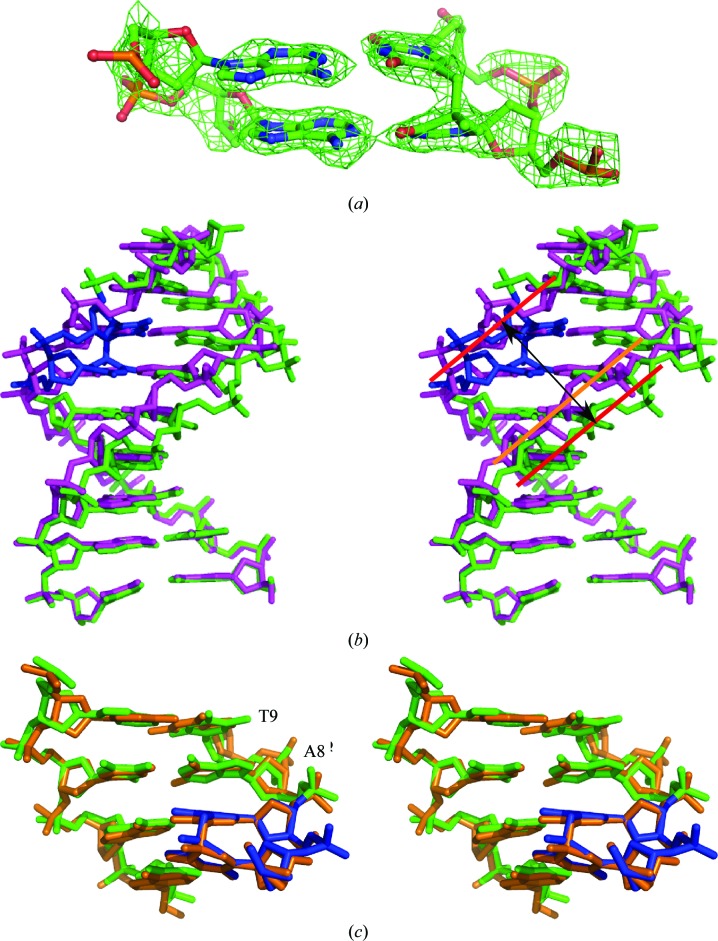
The SP lesion induces structural changes within the DNA. (*a*) The *F*_o_ − *F*_c_ electron-density map (green mesh) is shown prior to inclusion of the SP and complementary adenines in the structural model contoured at 2.5σ, with the final refined model for the lesion shown as a stick model. The electron density for the covalent bonding between the thymines is clearly evident. (*b*) Stick renderings of nine base pairs are shown in stereo for the SP-containing duplex DNA in green, the SP lesion in blue and the non-SP-containing DNA duplex in magenta. The first three base pairs within each structure superimpose well. Deviations in the two structures are then apparent beyond these first base pairs. The rough positioning of a spline through the phosphodiester backbone is indicated by red lines for the SP-containing duplex and by an orange line for the non-SP-containing duplex. In this view, the significant widening of the minor groove of the SP-containing DNA is apparent. (*c*). Stick renderings are shown in stereo for the base pairs (green) between the two SP lesions (blue) within our 16 bp oligonucleotide superimposed on the equivalent region of the structure of a single SP lesion (orange) found within an oligonucleotide complexed with *B. stearothermophilus* DNA polymerase I. The second SP lesion in our structure (not shown) would be on the strand complementary to that including A8 and T9.

The host–guest system is best suited to the analysis of symmetric DNA sequences, as 8 bp of the total 16 bp duplex are contained within the asymmetric unit, the unique repeating unit within the crystal (Goodwin *et al.*, 2005 ▶; Sun *et al.*, 1998 ▶). Thus, we analyzed the structures of two symmetric 16-base-pair duplexes, one with two SP lesions, one in each strand separated by two base pairs, and the other with the normal DNA of the same length and sequence (Figs. 1 ▶ *a*, 1 ▶ *b* and 1 ▶ *c*). Moreover, since the MMLV RT fragment alone was used as the search model to obtain molecular-replacement phasing, the initial electron density obtained for the guest DNA molecules was unbiased. Well defined electron density was observed for the SP lesion in initial electron-density maps (Fig. 2 ▶ *a*). Overall, the structures of the SP-containing and non-SP-containing DNA duplexes have clear structural differences, as shown in Fig. 2 ▶(*b*) and described in detail below.

### 3.2. Helical parameters   

The helical parameters and local base-pair geometries of the 16-base-pair SP-containing and non-SP-containing DNA sequences were computed using 3*DNA* (Colasanti *et al.*, 2013 ▶; Lu & Olson, 2003 ▶, 2008 ▶). The structural parameters associated with the SP structure may be influenced by the proximity of the two SP lesions, which are located within two base pairs of one another. However, the base pairs immediately adjacent to the SP lesions are very similar in structure to those found in the structure reported for a single SP lesion-containing structure in a complex with *B. stearothermophilus* DNA polymerase I (Fig. 2 ▶ *c*). In particular, the base pair immediately adjacent to the lesion, where both structures contain a purine in the position equivalent to A8, is nearly identical in the two structures. In the next base pair, the single SP lesion structure has a purine in the position equivalent to T9 but is still structurally quite similar. Thus, the proximity of the two lesions in our structure does not appear to cause structural changes that differ significantly from those induced by a single lesion.

**Figure 3:**
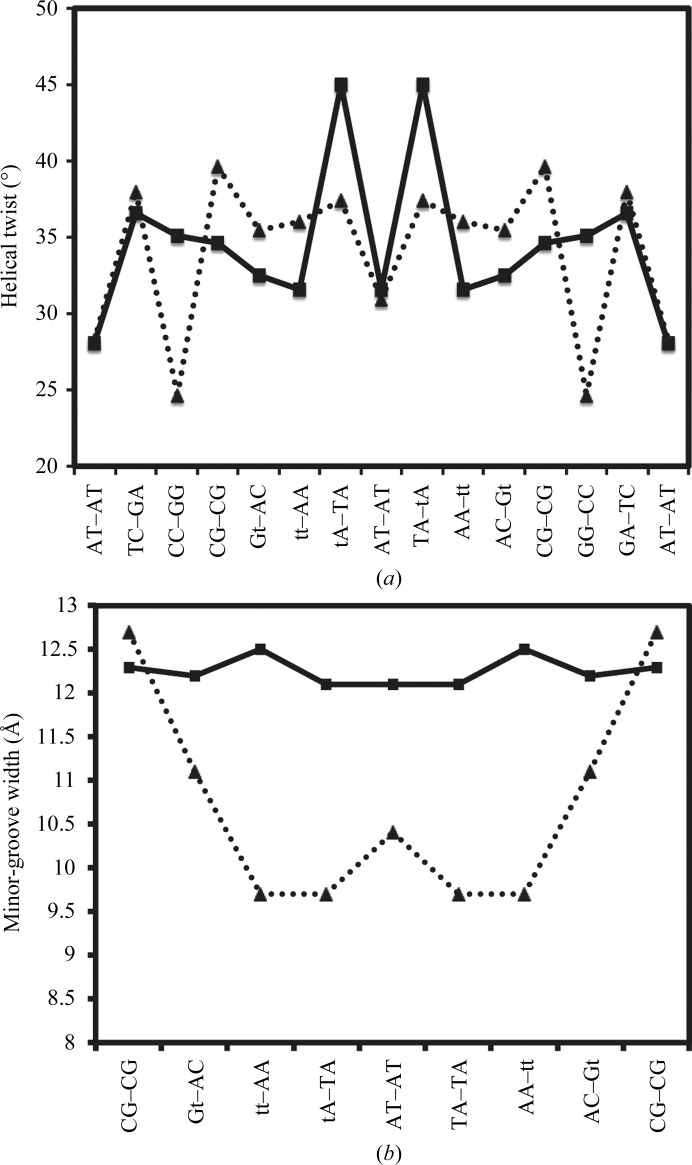
Comparative analysis of helical twist and minor-groove width in SP-containing and non-SP-containing DNA structures. (*a*) The change in helical twist for non-SP-containing (black triangles) and SP-containing DNA (black squares) are plotted with respect to base pairs. (*b*) Minor-groove widths are shown in the absence (black triangles) and presence (black squares) of the spore photoproduct lesion plotted for dinucleotide steps (bases 5–12).

Both structures maintain a right-handed DNA conformation with continuous base stacking and have average helical twists of 34.56° and 33.98°, corresponding to 10.4 and 10.6 bp per turn, respectively. However, the helical twist as a function of base pairs differs in the two structures, as shown in Fig. 3 ▶(*a*). A pronounced unwinding is observed at the t6t7 dinucleotide step (refer to Fig. 1 ▶ for the numbering scheme) in the region of the spore photoproduct lesion, where the DNA unwinds by an angle of −4.4° compared with the non-SP structure. However, the three dinucleotide steps following t6t7 show overwinding of the DNA, with values ranging from 0.68° to 7.6°. Collectively, these changes account for the accommodation of the spore photoproduct lesion in the double-helical DNA while maintaining Watson–Crick base pairing. Global helical twist values for the non-SP structure deviate significantly from those of the SP structure for dinucleotide steps C3C4 and C4G5, with values of 24.63° and 39.66° compared with 35.10° and 34.64°, respectively. Thus, these two steps are effectively underwound and overwound, respectively, in the non-SP structure compared with the SP structure.

The structure of the non-SP DNA is B-­form throughout, while that of the spore photoproduct shows three distinct regions in the DNA structure: the upper and the lower thirds adopt a B-form structure, while the central dinucleotide steps including the two spore photoproducts and intervening base pairs form some type of intermediate structure that deviates significantly from B-­form. The central region includes the dinucleotide steps tt/AA, tA/TA, AT/AT, TA/tA and AA/tt (B6–B11/G6–G11). The values of Zp for these dinucleotide steps along with helical inclination and *x* displacement, dimer step, roll and slide deviate substantially from those found in standard DNA conformations. The Zp and local base-pair parameters of the SP-containing and non-SP-containing DNA are listed in Table 2 ▶. A significant effect of the spore photoproduct lesion is also observed on the minor-groove width of the DNA. On comparing the minor-groove width of the SP-containing DNA with the non-SP form, it is clear that there is a significant widening of the minor groove (Fig. 3 ▶ *b*): the minor-groove width increases from 9.7 Å in the non-SP DNA to 12.5 Å in the SP-containing DNA. The widened groove is a result of changes in local base-pair parameters owing to the presence of the spore photoproduct, in which the two thymines are covalently linked to each other. The major-groove widths in the SP and non-SP structures are similar, with differences of less than 1 Å.

The most notable feature of the non-SP structure is that it has a very narrow minor groove ranging from 9.7 to 10.4 Å associated with its central TTATAA sequence. In this regard it differs from other AT-rich sequences that we have crystallized and analysed, in which the AATT sites were separated by a central GC pair (Glass *et al.*, 2009 ▶; Goodwin *et al.*, 2005 ▶, 2006 ▶). In these structures, the groove width was very narrow at the 5′-end of the AATT sequence and widened significantly towards the central GC pair.

### 3.3. Base-pair parameters   

The variations in the helical parameters and the groove widths of the SP-containing DNA from those of the non-SP form are a result of the effect of variations in the local base-pair step parameters as well as individual bases in response to the presence of the spore photoproduct. The SP-containing DNA suggests significant changes in the local base-pair step parameters of the t6t7 dinucleotide step of the spore photoproduct lesion. The tilt and twist for t6t7 reduce from 1.84° to −6.96° and from 35.79° to 25.49°, respectively. There is a significant increase in the value of roll from −3.60° in the non-SP-containing DNA to 17.52° in the SP-containing DNA (Table 2 ▶). A significant reduction is also observed in the case of *x* displacement (−0.58° to −5.49°) and global helical twist (36.01° to 31.61°) at t6t7. The decrease in tilt and twist and the reduced *x* displacement compared with the two adjacent base steps results in a widening of the minor groove. The reduced tilt of the bases to the helix axis also results in a propeller-like twist of the base pair, changing its value from −14.27° in the non-SP-containing DNA to −24.76° in the SP-containing DNA.

**Figure 4:**
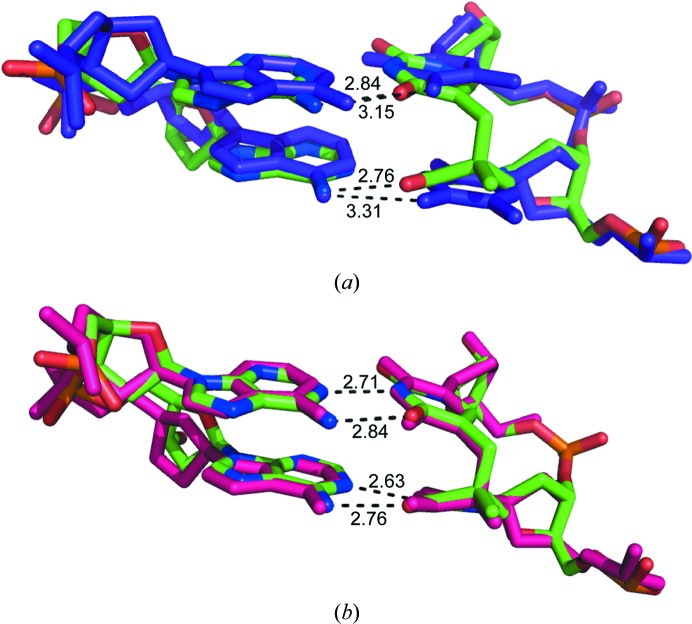
Comparison of TT–AA base pairs in SP-containing and non-SP-containing structures. (*a*) Stick renderings of superimposed non-SP TT–AA (purple) and SP tt–AA dinucleotide steps are shown. The hydrogen-bonding distances are shown for N6 of A11 and O4 of T6 for both base pairs in the dinucleotide step. For the non-SP-containing structure this hydrogen bond is very long at 3.31 Å, while in the SP-containing structure it is 2.76 Å. The hydrogen bonds between the same atoms in A10 and T7 are very similar. (*b*) Stick renderings of the SP-containing dinucleotide step in this work (C, green; N, blue; O, red; P, orange) and a mimic lacking the linking phosphate complexed with *B. stearothermophilus* DNA polymerase I (Heil *et al.*, 2011 ▶; pink) are shown superimposed. The structures are very similar despite the fact that the mimic lacks the linking phosphate. Hydrogen-bonding distances are shown for both base pairs of the SP structure and indicate strong hydrogen bonding of the SP lesion to complementary adenines.

Significant changes are also observed in the hydrogen-bonding distances of the base pairs involved in the formation of the SP lesion. t6 and t7 in the non-SP DNA have O4–N6 hydrogen-bond distances of 3.31 and 3.15 Å, respectively, compared with 2.76 and 2.84 Å in the SP DNA (Fig. 4 ▶ *a*). This suggests that the non-SP DNA has one long hydrogen bond within the A–T pair, while the corresponding A–t pair has more conventional hydrogen-bonding distances. Thus, the structure suggests that the hydrogen bonds among bases are predicted to be somewhat stronger in the SP-containing region. This is further supported by the decreased tilt, resulting in a significant twist in the SP lesion region. The normalized *B* factors for the SP-containing region or the equivalent region of the non-SP structure were within 15% of the average *B* factor and were similar in pattern to one another. Thus, the presence of the SP lesion does not appear to directly influence the dynamics of the structure as assessed by the *B* factors.

To determine whether the loss of aromaticity influences the stability of base stacking for t6, we analysed the contact area between t6 and G5 or t7 and compared this area with that in the non-SP-containing structure between T6 and G6 or T7 using *NACCESS* (Hubbard & Thornton, 1993 ▶). The contact areas between t6 and G5 or T6 and G5 were very similar in the two structures. However, the contact area calculated for t6 and t7 was actually slightly larger than for T6 and T7 (76 *versus* 67 Å^2^), suggesting that loss of aromaticity does not negatively impact base stacking in the SP lesion.

### 3.4. Main-chain and χ torsion angles   

The standard B-form DNA has glycosidic torsion angles (χ) in the *anti* conformation. Both the non-SP-containing and the SP-containing DNA have χ angles in the *anti* conformation except for G13 (− 86.1°, *syn*) in the SP-DNA. The change in the χ angle from −114.9° to −96.8° at t6 and from −104.9° to −121.9° at t7 in the SP-DNA reflects the structural change required to accommodate the spore photoproduct lesion. The backbone torsion angle β experiences the greatest deviation in the region of the spore photoproduct lesion, decreasing from 173.1° to −151.5° at t6 while increasing slightly from −175.6° to 174.5° at t7.

## 4. Discussion   

SP lyases are light-independent repair enzymes that specifically repair spore photoproduct lesions found in the spores of some bacteria such as *Bacillus* sp. (Friedel *et al.*, 2006 ▶). The important step in this repair mechanism is the recognition of the chemical structure of the lesion by the enzyme. Our crystal structures clearly show distortions induced by the lesion in the DNA double helix compared with the same sequence without the SP lesion, including loss of the B-conformation of the DNA in the region of the SP lesion, widening of the minor groove by approximately 3 Å and unwinding induced at the 5′-­T of the lesion followed by overwinding in the di­nucleotide steps following the lesion. An interesting and somewhat unexpected finding was that the hydrogen bonds from the T–T lesion to the corresponding As are not only maintained but are somewhat shorter, indicative of stronger bonds. The significant differences observed in DNA containing the SP lesion compared with other UV-induced lesions suggest a basis by which the SP lyase might recognize the lesion.

The 5*R*-SP lesion previously reported in the context of duplex DNA but lacking the central phosphate linkage (Heil *et al.*, 2011 ▶) is quite similar in structure to our SP lesion, as shown in Fig. 4 ▶(*b*). However, comparison of the SP-containing and non-SP-containing DNA duplexes crystallized in a complex with *B. stearothermophilus* DNA polymerase I reveals that the minor-groove widths for the two oligo­nucleotides are quite similar and are unusually wide: 13.6 and 14.1 Å, respectively, as analyzed in 3*DNA* (Colasanti *et al.*, 2013 ▶; Lu & Olson, 2003 ▶, 2008 ▶). The two DNA duplexes exhibit the A-form near the active site of the DNA polymerase, while the undamaged sequence is largely B-form and the SP-containing duplex is unclassified within the region of the SP. The unusual groove widths and helical forms exhibited by these DNA duplexes are undoubtedly influenced by inter­actions with the enzyme and thus differ significantly from our findings despite the structural similarity of the SP lesions themselves and the adjacent base pairs.

Compared with the SP lesion, which only shows widening of the minor groove, the cyclobutane pyrimide dimer (CPD) lesion shows widening of both the major and the minor grooves and an overall unwinding of 9.3°. Hydrogen bonding is lost between the 5′-thymine and its complementary adenine in CPD, while no such change is observed in the case of the SP lesion (Mees *et al.*, 2004 ▶; Park *et al.*, 2002 ▶). The other UV-induced lesions, which include (6–4) PD and Dewar lesions, show a greater distortion of the double helix compared with the CPD and SP lesions (Kim & Choi, 1995 ▶; Lee *et al.*, 1999 ▶). Nonetheless, the SP-containing DNA presents a unique surface and chemical structure compared with B-DNA that could facilitate recognition by the SP lyase repair enzyme.

Both the overwinding and underwinding in the region of the SP lesion and the significant widening of the minor groove observed in the crystal structure are in agreement with a number of proposed models for the recognition of UV-induced photoproduct lesions by lyases involving significant distortion of the DNA conformation caused by the lesions (Benjdia, 2012 ▶). Recently, the crystal structure of the SP lyase complexed with SAM and a spore photoproduct dinucleoside was reported (Benjdia *et al.*, 2012 ▶). Based on modeling of a substrate with the SP contained within a DNA duplex, it has been proposed that the lesion must be flipped out of the duplex in order to bind to the active site of the enzyme (Benjdia *et al.*, 2012 ▶), which would necessitate local melting of the DNA structure. This proposal is consistent with previous studies in which base flipping has been proposed for the repair of UV-induced photolesions (Yang *et al.*, 2013 ▶; Yang, 2011 ▶). Both CPD and (6–4) PD crystal structures with their respective lyases suggest weakening of the hydrogen bonds in the region of the lesion (Kim & Choi, 1995 ▶; Park *et al.*, 2002 ▶). As shown in thermal denaturation studies conducted by the Li laboratory, the presence of SP destabilizes the duplex oligonucleotide by 10−20 kJ mol^−1^ (Jian & Li, 2013 ▶). In assessing a structural basis for destabilization of the oligonucleotide induced by the presence of the SP lesion, we considered the following. Hydrogen-bonding distances between t–A base pairs are somewhat shorter, which is consistent with stronger hydrogen bonds. Loss of aromaticity of one thymine within the lesion does not appear to impact the stacking of the two bases as assessed by contact areas. Also, the overall dynamics within the lesion-containing region of the structure do not appear to differ significantly in the SP and non-SP structures as assessed by normalized *B* factors. Thus, these independent structural features within the SP structure do not provide an obvious explanation for the loss of stability. However, the global helical properties of the central region of the duplex containing the SP lesions differ significantly from those of the very stable B-form DNA, suggesting that collectively deviations from B-form DNA may be destabilizing. Moreover, the overall weakening effect of the lesion is likely to be the structural basis for the base-flipping mechanism during the SP repair process. Further experimental data are needed to clarify the mechanism by which the SP lesion might be flipped out of the duplex DNA and into the enzyme active site.

## Supplementary Material

PDB reference: N-terminal fragment of MMLV RT, SP DNA complex, 4m94

PDB reference: non-SP DNA complex, 4m95

## Figures and Tables

**Table 1:** Summary of crystallographic data and refinement statistics Values in parentheses are for the highest resolution shell. The refinement statistics were calculated using *PHENIX*.

Data set	SP†	Non-SP†
Data-collection statistics		
Unit-cell parameters ()		
*a*	53.96	54.33
*b*	145.72	146.10
*c*	46.88	46.97
Space group	*P*2_1_2_1_2	*P*2_1_2_1_2
Resolution range	33.732.14	26.701.72
Completeness (%)	93.70 (74.50)	98.20 (82.60)
*R*_merge_ (%)	4.74 (15.47)	4.90 (35.55)
*I*/(*I*)‡	21.70 (3.81)	24.36 (3.20)
Refinement statistics		
*R* value (%)	20.18	21.12
*R*_free_ (%)	23.95	23.40
R.m.s.d., bonds ()	0.006	0.004
R.m.s.d., angles ()	0.994	0.954
Average *B* factor (^2^)	29.53	34.98
Ramachandran plot statistics (%)		
Most favored	98	98.4
Additionally favored	2	1.6
Generously allowed	0	0

† SP, DNA containing the spore photoproduct lesion; non-SP, DNA lacking the spore photoproduct lesion.

‡ The average *I*/(*I*) values for the highest resolution shells are greater than 3.0, suggesting that the data extend to higher resolution. The data were cut at this resolution owing to incompleteness in the higher resolution shells.

**(a):** Base-pair step Zp ().

	SP†	Non-SP†
ttAA	0.20	0.03
tATA	0.58	0.30
ATAT	0.15	0.25
TAtA	0.58	0.30
AAtt	0.20	0.03

**(b):** Local base-pair parameters.

	ttAA, SP	TTAA, non-SP
Twist ()	25.49	35.79
Roll ()	17.52	3.60
Tilt ()	6.96	1.84
Shift ()	0.37	0.02
Slide ()	1.26	0.66
Rise ()	3.59	3.10

† SP, DNA containing the spore photoproduct lesion; non-SP, DNA lacking the spore photoproduct lesion.
